# Use of Contraceptives and Unmet Need for Family Planning among Tribal Women in India and Selected Hilly States

**Published:** 2014-06

**Authors:** Ranjan Kumar Prusty

**Affiliations:** International Institute for Population Sciences, Mumbai, India

**Keywords:** Scheduled tribes, Unmet need, Use of contraceptives, Central India

## Abstract

The paper provides a comprehensive picture of knowledge and use of contraceptives among scheduled tribes of India and selected central hilly states where tribal population contributes more than 30% of the total tribal population of the country. An attempt is also made to know how far scheduled tribes differ from non-tribes in the states, namely Jharkhand, Madhya Pradesh, and Chhattisgarh, using information collected in the third round of District-level Household Survey (DLHS-RCH III: 2007-2008). Bivariate analysis was used for understanding the level of knowledge, use of and unmet need for contraception among different tribal and non-tribal groups. Binary logistic regression was used for understanding the factors associated with the use of contraception and unmet need for family planning among tribal women. Knowledge and use of temporary contraceptive methods are considerably lower among tribal women compared to their non-tribal counterparts in the three states under study. Low acceptance due to phobia of adverse health consequences, accessibility to and lack of sound knowledge of contraception are the leading reasons for not using contraceptives. The unmet need for family planning among them was quite high, especially in the state of Jharkhand. Multivariate analysis substantiated the role of women and husbands’ education, age of women, and number of surviving boys in the use of any modern method of contraception. Educating women and their respective husbands about proper use and benefits of modern contraceptives is important to solve the problem of high unmet need for family planning among these tribal women. A simultaneous attention to the health systems strengthening component is crucial for ensuring sustained delivery of good-quality family planning services.

## INTRODUCTION

Ever since the family planning programme was introduced, India's demographic and health profile has changed radically (1). In the 1965-2009 period, contraceptive usage has more than quadrupled (from 13% of married women in 1970 to 56% in 2006), and the fertility rate has more than halved (from 5.7 in 1966 to 2.7 in 2006) but the national fertility rate is still high enough to cause long-term population growth. The United Nations estimated that world population grew at an annual rate of 1.23% during 2001-2010 whereas India's population grew at 1.64% per annum during 2001-2011 (2). Moreover, there exist large-scale variations and diversities in the demographic situation and socioeconomic and cultural milieu between and within the states and regions of the country. The fertility remains very high in most of the northern and central states. The contraceptive usage among these states is relatively lower than the southern and western states (3). There is also a high differential in contraceptive usage among different socioeconomic groups across the country.

Among the social groups in India, the tribes are the most socioeconomically-deprived groups, with low literacy and poor economic and living conditions (4). The tribal population, except some of the nomadic and denotified groups, is known as ‘scheduled tribes’ by Constitution of India. The scheduled tribes refer to various aboriginal ethnic minorities, who are basically concentrated in hilly lands—The Himalayas, Eastern Ghats, Western Ghats, Deccan Plateau, and other hilly regions of India. Tribal population in India constitutes 8.2% of the total population of the country (5). According to the National Family Health Survey (2005-2006), scheduled tribes in India have very high total fertility rate (3.12) than other social groups (3). In fact, there is a marginal increase in the total fertility rate which was 3.09 in the earlier round of the survey (1998-1999) and has gone unnoticed. Moreover, the tribes have a very low contraceptive usage and high unmet need for family planning than the other groups [Unmet need for contraception is the percentage of fertile married women of reproductive age, who do not want to become pregnant and are not using contraception].

Although there have been a burgeoning literature on various aspects of population, fertility, and family planning, there have been very few studies carried out among tribal population. A prominent study of access to family planning services among tribal population revealed the misconceptions and the problems of access associated with the usage of numerous methods (6). Another study carried out in three *talukas* of south Gujarat showed a dispersed pattern of attitude and acceptance in these regions (7). A micro-level study conducted on tribes in south India found low contraceptive usage and high unmet need for family planning among Adiya and Kattunayakan women in Kerala (8). The tribes have a very low rate of education, which makes them vulnerable to low contraceptive usage and high unmet need than other social groups. There is also ample literature at the national and international level, which substantiates the role of education in enhancing contraceptive use-rate (9-12). The NFHS survey (2005-2006) revealed that 52% of non-literate women did not use any contraceptive method while about a third of the non-literate fecund women not wanting a child did not use any contraceptive method.

The present paper tries to understand the behaviour relating to contraceptives among tribes in India and some of the selected high-fertility and low-literate tribal states in mid-Indian belt. Indian tribes are broadly classified into three main groups on the basis of race: Negrito, Proto-Australoid, and Mongoloid. The tribes belonging to Negrito group are negligible and are found in Andaman and Nicobar Islands and in the isolated pockets of Nilgiri district in South India. Mongoloid tribes are found in the northeastern states. They comprise only one-tenth of the total tribal population of the country and are socioeconomically better-off than the Proto-Australoid groups. The Proto-Austroloid groups are mostly distributed in the mid-Indian belt.

The mid-Indian belt constitutes around 70% of the total tribal population, out of which three states from central India, namely Jharkhand, Madhya Pradesh, and Chhattisgarh together comprise 30% of the tribal population of the country. These states have a high fertility rate and low literacy among women (Table 1). They are also lagging behind in many socioeconomic and demographic indicators than those in other states of the country. So, the present study focuses on differential in knowledge and use of contraceptives among tribal and non-tribal women. The study also examines several socioeconomic factors affecting the use of contraception and unmet need for family planning among the tribal women in these three states.

**Table 1.** Demographic profile of tribal and non-tribal population in selected states of India

Population/Women	Jharkhand	Chhattisgarh	Madhya Pradesh
Scheduled tribes (STs)#	7,087,068 (26.3)	6,616,596 (31.8)	12,233,474 (20.3)
Non-scheduled tribes (non-STs)#	19,858,761 (73.7)	14,217,207 (68.8)	48,114,549 (79.7)
% of India's tribal population	8.4	7.8	14.5
Total fertility rate†	3.30	3.10	3.12
Mean CEB tribal*	2.94	2.98	3.42
Mean CEB non-tribal*	2.93	2.86	3.01
Sex ratio (ST)#	987	1013	975
Literacy rate (tribal female)#	27.2	39.4	28.4
Total population	26,945,829	20,833,803	60,348,023

#Census 2001;

†NFHS 2007-2008;

*Mean no. of children ever born (CEB) calculated by author from DLHS 2007-2008 data

## MATERIALS AND METHODS

The information collected in the third round of District-level Health and Facility Survey (DLHS-RCH III: 2007-2008) is used in examining the level of knowledge, use of contraception, unmet need among tribal and non-tribal women of India and selected states. DLHS is a nationwide large-scale survey under Reproductive and Child Health (RCH) programme of the Government of India, which collects information on maternal and child health, family planning, and other reproductive health indicators. This survey collected information from 720,320 households, 643,944 ever-married women aged 15-49 years and 166,620 unmarried women aged 15-24 years covering 601 districts of 34 states and union territories of India (13). The survey was not conducted in the state of Nagaland. The dataset provides information about awareness and the use of family planning methods: female sterilization, male sterilization, intrauterine device (IUD), pill, emergency contraceptive pill (ECP), injectables, condom, female condom, rhythm method, and withdrawal method specified by currently-married women aged 15-49 years.

For the present analysis, four social groups, namely scheduled castes, scheduled tribes, other backward classes, and other castes from the datasets were categorized into two groups: ‘scheduled tribes’ are considered tribes, and ‘other caste groups’ are considered non-tribes. The ‘scheduled tribes’ and ‘tribes’ are used interchangeably in this study. The sample-size of the study women are shown in Table 2.

### Dependent variables

Information collected on contraceptive knowledge and current use of contraception has been used as dependent variables in the study. The major dependent variables used are as follows:

1. *Any method of contraception:* Those women using any of the contraceptive methods, both traditional and modern, are considered current users of any contraceptive method whereas women who are not using any of these methods are considered non-users.
2. *Any modern method of contraception:* A dichotomous variable (0=non-user, 1=user) is created using different modern methods of contraception: male sterilization, female sterilization, pill, IUD, condom, and injectibles. Those women using not any of these modern methods are considered non-users of modern method whereas any women using any of these methods are separated as current users of modern method.
3. *Unmet need for family planning*: Unmet need for family planning includes the proportion of currently-married women who are neither in menopause nor had hysterectomy nor are currently pregnant and who do not want any more children or want more children after two years or later and are currently not using any permanent or temporary methods of family planning. The women who are not sure about whether and when to have the next child are also included in unmet need for family planning. Unmet need is further categorized into ‘unmet need for spacing’ and ‘unmet need for limiting’ on the basis of temporary and permanent method respectively.

### Independent variables

Different social, economic and demographic characteristics of women are used as explanatory variables in the study. Sociodemographic variables taken into account are: age-group (15-19 years, 20-34 years, and 35 years and more), religion (Hindu/Christian/Others), place of residence (rural/urban), women's and husbands’ education in years (non-literate, <5 years, 5-9 years, and 10 or more years), Age at marriage in years (>18 years and <18 years), marital duration in years (<5 years, 5-9 years, 10-15 years, and >15 years), and number of surviving boys and girls. Economic indicator ‘wealth index’ constructed from household assets was used as an independent variable.

**Table 2.** Sample distribution of currently-married women aged 15-49 years in India (DLHS-3: RCH 2007-2008)

Social group	Jharkhand	Chhattisgarh	Madhya Pradesh	India
Scheduled tribes (%)	8,364 (32.5)	6,582 (38.9)	10,488 (23.7)	103,835
Non-scheduled tribes (%)	17,411 (67.5)	10,337 (61.1)	33,701 (76.3)	500,969
Total no. of women	25,775	16,919	44,189	604,804

Bivariate technique—cross-tabulation—was used for understanding differential levels of knowledge and the use of contraception among currently-married tribal and non-tribal women aged 15-49 years by different socioeconomic characteristics in India and selected states. Differentials in unmet need for spacing and limiting by socioeconomic and demographic factors were also examined through bivariate analysis. Binary logistic regression model was used for understanding the net effect of different socioeconomic and demographic factors on the current use of modern contraception and unmet need for family planning among tribal women. The mathematical equation for binary logistic regression is as follows:


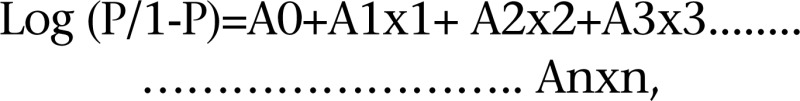


where Xi's are covariates and An's are coefficients. Dependent variables in the model used are: current use of any modern methods of contraception and unmet need for family planning. P and 1-P provide the odds ratios with respect to reference category; n is the number of predictors.

## RESULTS

### Knowledge of family planning methods

The knowledge of at least one contraceptive method was almost universal among women aged 15-49 years in India. However, among all methods of family planning, the awareness was little lower among the tribes than the non-tribes. The analysis reveals that knowledge of at least one method of contraception was almost identical among both tribal and non-tribal women. However, the knowledge of temporary contraceptive method was considerably low among tribal women compared to their non-tribal counterparts in India and all the three states. Only 60% tribal women were aware of condom compared to 78% of non-tribal women in India. Similar pattern was observed in all the selected states about awareness of different temporary methods: IUD, pill, ECP, injectables, and both male and female condoms. Among the officially-sponsored temporary methods, oral pill (76%) was the most popular modern temporary method among tribal women, followed by condom (60%) and IUD (56%). Among the states, the women (both tribes and non-tribes) of Jharkhand had relatively low knowledge of most contraceptive methods than the women of Madhya Pradesh and Chhattisgarh. Additionally, the level of knowledge about all contraceptive methods was very poor among tribes than the non-tribes in Jharkhand. In the states under study, only 31% tribal women were aware of male condom compared to more than 45% of non-tribe women (Table 3).

**Table 3.** Knowledge of different contraceptive methods (in percentage) among currently-married tribal and non-tribal women (15-49 years) in India and selected states

Method	Jharkhand		Chhattisgarh		Madhya Pradesh		India	
	Tribe	Non-tribe	Tribe	Non-tribe	Tribe	Non-tribe	Tribe	Non-tribe
Any method	88.6	95.5	99.3	99.7	97.3	99.3	97.0	99.4
Any modern method	86.5	95.2	99.2	99.6	97.2	99.2	96.5	99.3
Permanent methods								
Female sterilization	84.7	94.0	98.9	99.3	96.8	98.9	94.6	98.6
Male sterilization	55.4	70.5	88.6	91.9	78.3	87.9	68.8	85.6
Modern temporary methods							
IUD	26.9	41.4	35.6	60.6	33.6	63.6	56.3	77.4
Pill	55.8	69.2	75.0	86.6	62.0	84.6	76.1	88.0
ECP	8.9	16.8	7.3	19.9	15.0	33.1	20.8	33.3
Injectables	14.0	27.7	16.2	33.8	30.2	56.0	31.4	56.9
Male condom	30.7	45.7	56.6	74.2	41.5	71.5	60.2	77.8
Female condom	5.0	10.1	3.5	16.4	3.7	10.3	8.3	13.1
Traditional methods								
Rhythm/Abstinence	21.4	24.9	23.9	36.7	27.9	43.1	40.1	55.8
Withdrawal	11.9	13.7	20.6	28.9	19.1	32.6	33.3	41.8
Other	12.0	3.7	11.8	6.3	1.8	1.1	3.3	1.8

ECP=Emergency contraceptive pill

### Current use of family planning methods

Although all women knew at least one contraceptive method, only a half of them were using any contraceptives in India. Current contraceptive prevalence among currently-married tribal women was relatively lower—only 45% and 39% compared to 53% and 47% of the non-tribal women using any method and any modern method of contraception respectively. However, the difference in the selected states was wider among the two social groups (Table 4). In Jharkhand, only 17% of the tribal women used any modern method of contraception compared to 39% of the non-tribes. Majority of these tribal women, like their non-tribal counterparts, preferred female sterilization (28%). Only 2% tribal couples used condoms against 5% non-tribal couples. The condom usage was low among both tribes and non-tribes in the selected states. However, the condom usage was lower among tribal women than non-tribal women. A wide gap between knowledge and practice of family planning was noticed both among scheduled tribe and non-tribe women. However, the gap was relatively wider for tribal women as about 97% tribal women were aware of female sterilization but less than one-third of them were using female sterilization.

### District-level variation in contraceptive usage

**Figure 1.**
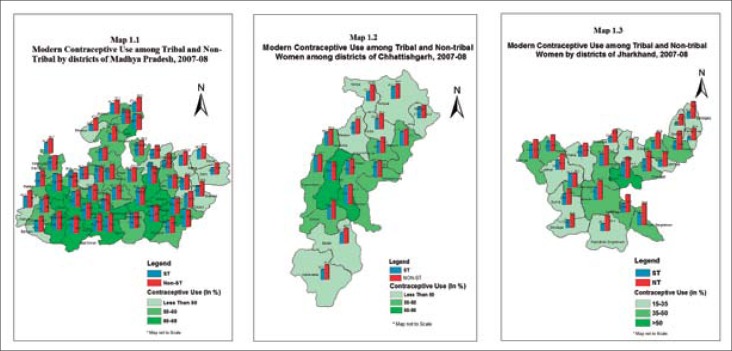
Map showing the district-level variation in contraceptive usage

District-level variation in modern contraceptive usage is shown in Figure 1 (map). Madhya Pradesh is located in central India whereas Chhattisgarh is situated towards the east and Jharkhand in the northeast of Madhya Pradesh. The proportion of contraceptive usage ranged from 16% in Pakaur district of Jharkhand to 68% in Damoh district of Madhya Pradesh. In most of the districts of Madhya Pradesh and central districts of Chhattisgarh, more than half of the women used any modern method of contraception whereas, in Jharkhand, 21 of the 22 districts (except Bokaro district) had contraceptive prevalence rate lower than 50%. Interestingly, five districts in north of Chhattisgarh and five districts in northwest of Madhya Pradesh situated near Jharkhand had a low contraceptive rate of less than 50%. Dantewada (38%) and Baster (40%), the two southern districts of Chhattisgarh which have mostly been in news due to Naxal activities had also lower CPR among the women than the central districts of Chhattisgarh. Of the total 83 districts in the three selected states, 74 districts had lower contraceptive prevalence among the tribal women than the non-tribal women. The widest difference is observed in Sahibganj, Dumka, Purbi Singhbhum, Saraikela Jamtara (all in Jharkhand), Shivpuri, Panna, Sheopur, Jamatara (all in Madhya Pradesh), and Koriya (in Chhattisgarh) districts of the three states.

### Socioeconomic differentials in contraceptive usage

The tribal and non-tribal differentials in the use of any modern contraceptives by socioeconomic category have been shown in Table 5. Among the tribal women, there was high differential in contraceptive usage among different socioeconomic groups. For instance, only 36% of Christian tribal women used modern contraceptives compared to 45% of Hindu tribal women in India. Moreover, just 34% women belonging to the poorest categories of wealth quintile used any modern contraceptive compared to 54% women belonging to the richest categories of wealth quintile. The differences were wider in the state of Jharkhand by almost all socioeconomic groupings. In Jharkhand, only 15% tribal women without any education used modern contraceptives compared to 35% of women with 10 or more years of education. By economic stratum, merely 13% of women from the poorest stratum used modern contraceptives compared to 52% of women belonging to the richest stratum in Jharkhand.

**Table 4.** Current use of different contraceptive methods (in percentage) among currently-married tribal and non-tribal women (15-49 years) in India and selected states

Contraceptive method	Jharkhand		Chhattisgarh		Madhya Pradesh		India	
	Tribe	Non-tribe	Tribe	Non-tribe	Tribe	Non-tribe	Tribe	Non-tribe
Any method	22.8	41.8	43.5	56.2	50.2	60.2	44.6	53.3
Any modern method	17.2	38.8	40.1	54.2	48.0	57.0	39.5	46.8
Female sterilization	11.6	32.5	33.8	48.5	44.3	48.0	27.8	34.2
Male sterilization	0.3	0.5	3.8	1.3	1.5	0.9	1.9	0.8
IUD	0.6	0.5	0.2	0.8	0.1	0.6	1.8	1.5
Pill	3.0	2.8	1.6	1.4	0.8	1.9	4.3	3.2
ECP	0.3	0.2	0.1	0.1	0.1	0.2	0.2	0.1
Condom	1.3	2.2	0.5	2.0	1.1	5.2	1.8	5.1
Rhythm	2.6	1.9	1.1	0.9	1.4	2.3	3.4	4.6
Withdrawal	0.5	0.5	0.4	0.4	0.6	0.8	1.5	2.0

**Table 5.** Current use of any modern method of contraception (in percentage) among currently-married women (15-49 years) in selected states by background characteristics, 2007-2008

Background characteristics	India		Jharkhand		Chhattisgarh		Madhya Pradesh	
	Tribe	Non-tribe	Tribe	Non-tribe	Tribe	Non-tribe	Tribe	Non-tribe
Religion								
Hindu	45.2	51.1	18.1	42.8	40.2	54.4	48.0	57.2
Christian	36.5	53.7	15.9	15.0	41.0	55.4	46.6	72.5
Other	38.1	41.2	16.9	18.4	21.1	44	71.0	53.6
Residence								
Rural	40.6	46.9	16.1	35.7	39.9	53.1	47.7	56.4
Urban	51.7	54.0	36.8	52.7	45.8	57.5	53.0	58.5
Education								
Non-literate	42.3	47.1	15.2	36.0	42.5	57.6	49.9	60.7
Less than 5 years	41.4	54.5	16.3	37.1	42.1	58.9	49.5	61.1
5-9 years	41.1	50.8	19.6	41.4	31.7	48.0	37.4	51.3
10 or more years	43.2	50.0	35.3	47.7	40.0	52.1	41.7	53.4
No. of surviving girls								
0	27.2	36.0	10.4	26.5	24.6	35.7	28.3	40.5
1	48.5	57.4	19.8	45.0	44.3	61.0	56.5	65.4
2+	48.7	53.9	21.0	43.7	50.6	64.0	55.3	64.1
Wealth index								
Poorest	33.6	34.2	12.9	27.7	33.5	43.8	44.2	50.0
Second	39.8	41.0	20.2	35.6	45.5	52.0	49.8	54.4
Middle	44.1	49.3	29.1	42.7	48.6	55.7	54.8	58.2
Fourth	51.2	53.1	37.3	49.6	49.8	60.3	55.3	59.0
Richest	54.6	56.5	52.6	56.6	52.3	61.5	65.4	62.7
Total	42.0	49.4	17.2	38.8	40.1	54.2	48.0	57.0

### Main reasons for not using modern contraception

The main reasons for not using contraception among tribal women are shown in the Table 6. Self-resistance to use was the leading reasons cited by tribal women of Jharkhand (28%) and Chhattisgarh (29%). The self-opposition to use contraception might be due to health concerns and lack of proper knowledge about sterilization in the above two states as there were very higher proportion of tribal women who explained health concern, fear of side-effects, scared of being sterilized, and thinking that they cannot work after sterilization as important reasons for not using sterilization compared to tribal women in Madhya Pradesh. In Jharkhand, 11% tribal women who did not use any contraception told they did not know any method of contraception. In addition, more than 5% of the women expressed they did not know the source from where to get these or there was no access to contraception for them. The other leading reasons for not using contraception conveyed by the tribal women of the three states were infrequent/no sex, menopause, breastfeeding, and postpartum amenorrhoea. A huge proportion of women also believed, fertility is up to God, and they should not restrict it through contraception.

#### Multivariate analysis

Use of modern methods among couples in the selected states were affected by many factors, like age of women, religion, education of the couples, marital duration, number of surviving boys and girls, and economic status. Table 7 describes the odds ratios for the use of any modern contraceptive methods among tribal women in the study states. Results reveal that women with higher age, higher education, higher number of children, higher duration of marriage were more likely to use any modern method of contraception than the younger women (15-24 years). Use of any modern contraceptive methods were found more likely among older women than younger ones in all the study states, except Jharkhand, which did not show a significant relationship. Higher-educated women were significantly more likely to use any modern contraceptive methods than the non-literate women of all the three states. Women reporting of having more than two surviving boy children were more likely to use any modern contraceptive methods than those woman having any surviving boy child. Women who reported of having more than two surviving girl children were less likely to use any modern contraceptive methods, and both of the cases showed a significant relationship with the use of modern contraceptives. With the increasing wealth quintile, the use of any modern contraceptives was also growing from the poorest to the richest group in all selected states. For example, the richest tribal women in Jharkhand were 4.5 times more likely to use contraception than women belonging to the poorest categories.

**Table 6.** Reasons for not using modern contraception among tribal women in the selected states

Main reason	Jharkhand	Chhattisgarh	Madhya Pradesh
Fertility-related			
Infrequent sex, no sex, husband away	12.3	9.6	10.2
Menopause, hysterectomy	5.7	4.5	6.4
Sub-fecund, in-fecund	0.2	0.8	0.6
Breastfeeding/Postpartum amenorrhoea	14.2	15.9	20.2
Up to God	13.5	21.7	16.7
Opposition to use			
Respondent opposed	27.7	29.1	15.2
Husband opposed	3.1	6.6	2.5
Others opposed	0.2	1.3	0.5
Religion prohibited	0.5	0.4	0.1
Lack of knowledge			
Knows no method	10.6	1.6	5
Knows no source/lack of access	5.3	2.4	2.6
Method-related			
Health concerns	12.3	10.8	4.1
Fear of side-effects	2.6	2	0.3
Cost too much	2.6	1.5	1.4
Interferes with body	0.9	0.6	0.8
Afraid of sterilization	10.0	9.1	5.1
Cannot work after sterilization	4.4	1.6	2.3

**Table 7.** Logistic regression showing odds ratios for any modern methods of contraception by different socioeconomic and demographic variables of currently-married tribal women in the selected states

Background characteristics	Any modern method of contraception		
	Jharkhand	Chhattisgarh	Madhya Pradesh
	[Exp (β)]	[Exp (β)]	[Exp (β)]
Age-group (completed years)			
15-19®			
20-34	1.36	1.83**	1.96***
35 and more	1.26	2.02**	2.09***
Religion			
Hindu®			
Christian	0.52***	0.81	0.70
Other	0.85**	0.42	3.31**
Residence			
Rural®			
Urban	1.17	0.84	0.81*
Education			
Non-literate®			
Less than 5 years	1.09	1.36***	1.45***
5-9 years	1.43***	1.37***	1.22**
10 or more year	2.45**	1.62**	1.44*
Husband's education			
Non-literate®			
Less than 5 years	1.09	1.22**	1.25***
5-9 years	1.28***	1.22**	1.32***
10 or more years	1.41***	1.15	1.33***
Marital duration			
Less than 5®			
5 to 10 years	1.39**	1.58***	2.60***
10 to 15 years	2.28***	3.28***	6.43***
15 years	3.30***	5.13***	9.00***
Age at marriage			
>18 years®			
<18 years	0.99	0.93	1.26***
No. of surviving boys			
0®			
1	2.92***	5.90***	5.01***
2+	5.17***	12.77***	12.54***
No. of surviving girls			
0®			
1	1.52***	1.75***	2.22***
2+	1.39***	1.81***	1.48***
Wealth index			
Poorest®			
Second	1.64***	1.89***	1.43***
Middle	2.43***	2.33***	1.84***
Fourth	3.11***	2.47***	2.04***
Richest	4.50***	2.29***	3.41***
-2 Log likelihood	6530.20	6558.89	10632.62

®Reference category;

***1% significance level;

**5% significance level;

*10% significance level

### Unmet need for family planning

**Figure 2.**
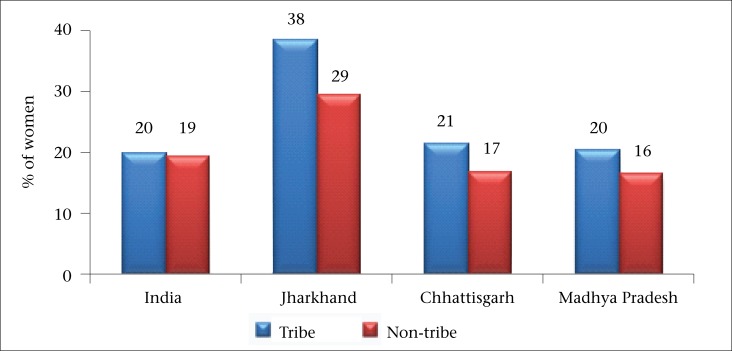
Unmet need for family planning among tribes and non-tribes in India and selected states

Recognition of unmet need for family planning is important for achieving demographic goals of below-replacement fertility. Results proved that, in unmet need for family planning, there was little difference between tribes and non-tribes in India. However, in the selected states, a huge gap was observed in unmet need for both spacing and limiting methods of contraception (Table 8). The tribal women had a very high unmet need for contraception compared to those in Jharkhand (Figure 2).

### Socioeconomic differentials in unmet need for family planning

Unmet need for family planning was very high among tribal women than the non-tribes by almost all socioeconomic indicators (data not shown). Wide differences in unmet need for both spacing and limiting was observed between different socioeconomic categories, like age of women, marital duration, age at marriage, and number of surviving boys. Among the tribal women, total unmet need was higher among the less-educated, rural and Hindu women than the higher-educated, urban, Christian women. The unmet need for spacing was higher among the younger women (15-19 years), recently-married (marital duration less than 5 years), and non-literate tribal women whereas unmet need for limiting method was higher among older women, those married for more than 10 years, and higher-educated tribal counterparts (data not shown). Overall, unmet need for limiting methods was much higher than spacing method of family planning in Jharkhand and Chhattisgarh.

#### Multivariate analysis

Table 9 shows the odds ratios of logistic regression analysis of unmet need for family planning methods among tribal women in the study states. A number of explanatory variables, such as women's age, marital duration, economic condition, and number of living children, have been found to be statistically significant determinants of unmet need for family planning in the selected states. With increase in marital duration, the women were significantly less likely to have unmet need for family planning. For example, the tribal women with marital duration of 15 years or more was 76% and 79% less likely to have unmet need for contraception in Jharkhand and Madhya Pradesh respectively. Similarly, economic status (wealth index) and unmet need show an inverse relationship. The richest women were less likely to have unmet need for contraception than the poorest women in all three states. The unmet need for family planning methods was significantly and positively associated with number of surviving boys and girls in all three states. Women aged 35 years or more had significantly lower unmet need for family planning than the younger women in between 15 and 19 years in Jharkhand (OR=0.64, p<0.05) and Madhya Pradesh (OR=0.55, p<0.01).

**Table 8.** Unmet need for spacing and limiting methods among currently-married tribal and non-tribal women (15-49 years) in India and selected states

Unmet need	India		Jharkhand		Chhattisgarh		Madhya Pradesh	
	Tribe	Non-tribe	Tribe	Non-tribe	Tribe	Non-tribe	Tribe	Non-tribe
Spacing	7.5	6.6	13.2	11.7	8.9	7.1	9.3	6.8
Limiting	12.3	12.5	24.8	17.5	12.4	9.6	10.8	9.5

## DISCUSSION

The focus of the paper was on the contraceptive usage in hilly states of India where fertility level was very high. The states have inaccessible terrain, and settlements are scattered over a vast area, which possess several formidable problems to family planning and reproductive health delivery system (14). In these states, the study revealed that almost all tribal women knew at least one family planning method (modern or traditional); however, the knowledge of temporary methods was relatively poor among them. The findings of the study are similar to many other micro-level studies carried out among tribes of India (15,16). The low literacy status and limited availability of mass media, such as radio and TV in tribal areas also play a role in poor awareness of temporary contraceptive methods (17). The high endorsement of female sterilization promotes higher knowledge and use of sterilization. Although the Government has recently shifted away from its policy of promoting female sterilization as the main method of family planning, the health service providers has not come up with promoting other temporary methods of contraception (18). The main reason explained by several studies is that sterilization requires one-time motivation whereas the motivation for the spacing methods requires sustained efforts (19).

The wide gap between knowledge and the use of contraception was observed, and the gap was significantly wider for tribal women than that for non-tribal women. The present study showed that only about half of them were using any contraceptive methods in the three selected states. The contraceptive prevalence among tribal women was relatively lower, with only 40% of them using modern contraceptives compared to 47% of the non-tribal women. The gap was observed to be very much wider in the state of Jharkhand than in Madhya Pradesh and Chhattisgarh. The family planning programme in Jharkhand failed to reach the poorest section of the society, which had very low contraceptive usage than the richest group (20). In Jharkhand, low acceptance, accessibility to and lack of sound knowledge of contraception were the leading reasons for not using contraceptives. The self-opposition and phobia for adverse consequences to health were other leading causes coming out of the analysis.

**Table 9.** Logistic regression showing odds ratios of unmet need for contraception by different socioeconomic and demographic variables of currently-married tribal women in the selected states

Background characteristics	Unmet need for contraception		
	Jharkhand	Chhattisgarh	Madhya Pradesh
	[Exp (β)]	[Exp (β)]	[Exp (β)]
Age (completed years)			
15-19®			
20-34	0.92	0.93	0.99
35+	0.64**	0.92	0.55***
Religion			
Hindu®			
Christian	1.08	1.20	1.24
Other	1.12*	2.60*	0.22
Residence			
Rural®			
Urban	1.10	1.23	1.19
Education			
Non-literate®			
Less than 5 years	1.28*	0.87	0.91
5-9 years	1.11	0.98	1.04
10 or more years	0.83*	1.12	1.32
Husband's education			
Non-literate®			
Less than 5 years	0.99	0.97	0.84*
5-9 years	0.98	1.03	0.91
10 or more years	1.01	1.06	0.98*
Marital duration			
Less than 5 years®			
5 to 10 years	0.48***	0.85*	0.57***
10 to 15 years	0.33***	0.89	0.28**
15 years and above	0.24***	0.74	0.21**
Age at marriage (years)			
>18®			
<18	1.07	1.01	0.95*
No. of surviving boys			
0®			
1	3.29*	2.53***	2.26***
2+	4.88*	3.10***	1.68***
No. of surviving girls			
2+	3.64***	3.83***	2.58***
Wealth index			
Poorest®			
Second	0.83***	0.70***	0.81***
Middle	0.68***	0.60***	0.82***
Fourth	0.61**	0.63**	0.66**
Richest	0.41**	0.58*	0.44**

®Reference category;

***1% significance level;

**5% significance level;

*10% significance level

Among users of family planning methods, more than 80% tribal couples were using a non-reversible method. The female sterilization alone contributed more than three-fourths of the total contraception usage, which suggests that tribal women were mainly using family planning methods to limit their family-size, and spacing of children was quite neglected. The higher acceptance of sterilization among the tribes was due to their poor economic condition and the financial incentives associated with sterilization (21). Unsystematic ways of motivation for spacing methods by health workers and lack of awareness about various family planning methods among the tribal women could be contributing factors for their heavy reliance on sterilization. Many micro-level studies conducted among the most backward primitive scheduled tribes of central India also revealed that, despite Government's ban on sterilization among primitive tribes in 1979, monetary issues were the most important reasons for higher acceptance of sterilization in these groups (15).

The National Population Policy insists the Government's commitment to the provision of quality service, information and counselling, and expanding contraceptive method choices in order to enable people to make voluntary and informed choices (22). Still there is very high unmet need for family planning, and it is very much higher among the tribal women than the non-tribal women. Among the states, the gap was very much evident in Jharkhand both in terms of unmet need for spacing and limiting methods. Moreover, there were intra-tribal inequalities by socioeconomic indicators. Rural and women belonging to the poorest and poorer wealth quintiles had a very high unmet need compared to the urban and the richest women. Younger women had a very high unmet need for spacing method whereas older women had a very high unmet need for limiting method of contraception. The question, again, can be emphasized on access to different contraceptive methods. Most rural and poor tribal women were confined within the underdeveloped region where health facilities are still in underprivileged states.

From the multivariate analysis, it can be summarized that education of both tribal women and their respective husbands, number of surviving boys and girls, and economic characteristics of the household the women belonged to, had significant and positive impact on the use of both any method and a modern method of contraception. The higher-educated tribal couples (considering both women's education and husbands’ education, which were independently used in the logistic regression model) were more likely to use any method of contraception than the non-literate couples, which has been proven in many researches on general population. The analysis also proves that women having two or more male children have very high odds ratios of using contraception than women having two or more girl children. So, it can be concluded that tribal women in the three states had very high son preferences, which was louder among tribal women from the state of Madhya Pradesh and Chhattisgarh. This needs further research as most studies show that tribal couples have low son preferences. The Christian tribal women were less likely to use any modern method of contraception than the Hindu women in the state of Jharkhand. Religion shows no significant association with contraception in both central Indian states—Madhya Pradesh and Chhattisgarh. The richest women were more likely to use contraception and less likely to have unmet need for contraception than the poorest women in all the three states.

### Conclusions

It can be concluded that the contraceptive usage and unmet need remain substantially high among tribal women in all selected states. In Jharkhand, a very high difference in contraceptive usage among tribal and non-tribal currently-married women was observed. Improvement in the contraceptive prevalence rate and addressing the unmet need for contraception require sustained efforts by health workers to ensure quality care to the beneficiaries. The most important is: improving literacy among the tribal couples that would significantly contribute to these efforts. Focus on improving information, education and communication (IEC) activities is the key to addressing the unmet needs for contraception, along with easily-accessible, convenient, and good-quality methods of family planning. Role of husbands needs to be strengthened as it is found that husbands’ education plays a significant role in contraceptive usage.

It is important to focus on high unmet need for family planning and contraceptives among tribal women in the state, with an inclusive policy focusing on its poorest section. If the goal is to create a demand for adoption of family planning and services, a check in the potential future unmet category is needed. A simultaneous attention to the health systems strengthening component is crucial for ensuring sustained delivery of good-quality services. Development of the family planning strategy is an important milestone and should be followed up with implementation, resource allocation, and equity-based monitoring and evaluation.

## ACKNOWLEDGEMENTS

The author is grateful to Prof. Sayeed Unisa for her constructive comments and suggestions on various sections of the paper. Authors would also like to thank the editors and two anonymous reviewers for their suggestions towards improvement of the paper.
